# Pullback Bundles and the Geometry of Learning

**DOI:** 10.3390/e25101450

**Published:** 2023-10-15

**Authors:** Stéphane Puechmorel

**Affiliations:** ENAC (École Nationale de l’Aviation Civile), Université de Toulouse, 7, Avenue Edouard Belin, 31055 Toulouse, France; stephane.puechmorel@enac.fr

**Keywords:** pullback bundle, information geometry, machine learning

## Abstract

Explainable Artificial Intelligence (XAI) and acceptable artificial intelligence are active topics of research in machine learning. For critical applications, being able to prove or at least to ensure with a high probability the correctness of algorithms is of utmost importance. In practice, however, few theoretical tools are known that can be used for this purpose. Using the Fisher Information Metric (FIM) on the output space yields interesting indicators in both the input and parameter spaces, but the underlying geometry is not yet fully understood. In this work, an approach based on the pullback bundle, a well-known trick for describing bundle morphisms, is introduced and applied to the encoder–decoder block. With constant rank hypothesis on the derivative of the network with respect to its inputs, a description of its behavior is obtained. Further generalization is gained through the introduction of the pullback generalized bundle that takes into account the sensitivity with respect to weights.

## 1. Introduction

Explainable Artificial Intelligence (XAI) is generally described as a collection of methods allowing humans to understand how an algorithm is able to learn from a database, reproduce and generalize. It is currently an active, multidisciplinary area of research [1,2] that relies on several theoretical or heuristic tools to identify salient features and indicators explaining the surprisingly performances of machine learning algorithms, especially deep neural networks. From a statistical point of view, a neural network is nothing but a parameterized regression or classification model, that can be described as a random variable whose probability distribution is known conditionally to external inputs and internal parameters [3]. Unfortunately, even if this approach seems the most natural one, it is not adapted to XAI as no insight is gained on the learning and inference process. Furthermore, it seems that there is a contradiction between the statistical procedure that appeals for models with the smallest possible number of free parameters and the performance of deep learning relying on thousands to millions weights. On the other hand, attempts have been made to design numerical [4] or visual [5] indicators aiming at producing a summary of salient features.

XAI is also related to acceptable AI, that is proving or at least ensuring with a high probability that the model will produce the intended result and is robust to perturbations, either inherent to the data acquisition process or intentional. In both cases, it is mandatory to be able to perform a sensitivity analysis on a trained network. In [6], an approach based on geometry was taken and the need of a metric on the set of admissible perturbations enforced. The problem of the so-called adversarial attacks is treated in several papers [7,8,9] where mitigating procedures are proposed. Adversarial attacks are a major concern for acceptable AI, especially in critical application like autonomous vehicles or air traffic control. From now, most of the research effort was dedicated to the design of such attacks with the idea of incorporating the fooling inputs in the learning database in order to increase robustness. The reader can refer, for example, to Fast Gradient Sign methods [10], robust optimization methods [11] or DeepFool [12,13]. Unfortunately, while these approaches are relevant to acceptable AI, they do not provide XAI with usable tools. Furthermore, they rely on inputs in $R^{n}$, or generally in a finite dimensional Euclidean space, which is not always a valid hypothesis.

There is also a question on why learning from a high dimension data space is possible, and a possible answer is because data effectively lies on a low dimensional manifold [14,15]. As a consequence, most of the directions in the input space will have a very small impact on the output, while only a few number of them, namely those who are tangent to the data manifold, are going to be of great influence [16]. The manifold hypothesis also justifies the introduction of the encoder–decoder architecture [17] that is of wide use in the field of natural language processing [18] or time-series prediction [19]. The true underlying data manifold, if it exists, is most of the time not accessible, although some of its characteristics may be known and incorporated in the model. In particular, it may be subject to some action by a Lie group or possess extra geometric properties, like the existence of a symplectic structure. Specific networks have be designed to cope with such situations [20,21].

In a general setting, little is known about the data manifold and its geometric features, like metric, Levi-Civita connection and curvature. However, Riemannian properties are the most important ones as they dictate the behavior of the network under moves in the input space. Recalling the statistical approach invoked before, it makes sense to model the output of the network as a density probability parameterized by inputs and weights. Within this frame, there exists a well-defined Riemannian metric on the output space known as the Fisher Information Metric (FIM) originating from a second order expansion of the Kullback–Leibler divergence. The importance of this metric has already been pointed out in several past works [22,23]. The FIM can be pulled back to the input space, yielding, in most cases, a degenerate metric that can nevertheless be exploited to better understand the effect of perturbations [16], or to parameter space to improve gradient-based learning algorithms [24]. In this last case, however, things tend to be less natural than for the input space.

In this work, a unifying framework for studying the geometry of deep networks is introduced, allowing a description of encoder–decoder blocks from the FIM perspective. The pullback bundle is a key ingredient in our approach.

In the sequel, features and outputs are random variables, thus characterized by their distribution functions, or their densities in the absolutely continuous case. Within this frame, a neural network is a random variable:

(1) $$\left\{\begin{array}{c} Y=N\left(X,W\right)\\ X:\left(\Omega ,T,P\right)\to \left(E,E\right)\\ W:\left(\Omega ,T,P\right)\to \left(\Theta ,F\right) \end{array}\right.$$

where $\left(\Omega ,T,P\right)$ is an underlying probability space and $\left(E,E\right),\;\left(\Theta ,F\right)$ are, respectively, the input and weight measure spaces Finally, *Y* is assumed to take its values in the output measure space $\left(O,O\right).$ Most of the time, the network has a layered structure so that the expression of N can be factored out as:

(2) $$Y=N\left(N\left(\ldots ,W_{2}\right),W_{1}\right)$$

In many practical implementations, the weights *W* are deterministic, that is equivalent to saying that their probability distribution is a Dirac distribution. In this case, a neural network can be described as a parameterized family of random variables $N_{W}:\omega \mapsto N\left(X(\omega ),W\right)$. A special case occur when a single decoder is considered [25], that is, a measurable function:

(3) $$f=N\left(\cdot ,W\right):R^{d}\to R^{m},d\leq m$$

where *f* is a smooth mapping, assumed in [25] to be an immersion; that is, for any *x*, $Df_{x}$ has maximal rank d. Conversely, one may consider an encoder

(4) $$g=N\left(\cdot ,\tilde{W}\right):R^{n}\to R^{d},\;d\leq n$$

and assume *f* to be a submersion. In this paper, the geometry of the complete encoder–decoder network

(5) $$g\circ f=N\left(N\left(\cdot ,W\right),\tilde{W}\right)$$

will be considered, as well as the case d≥m, d≤n.

The article is structured as follows: In Section 2, the Fisher information metric is introduced and some formulas, valid when the parameter space is a smooth manifold, are given. In Section 3, the pullback bundle is defined and applied to the encoder–decoder case. Finally, a conclusion is drawn in Section 5. The convention of summation on repeated indices applies in this manuscript.

## 2. The Fisher Information Metric

In this section, we recall some basic definitions and properties in information geometry. The foundational ideas can be traced back to [26], but the main developments occur quite recently. The reader is referred to [27] for a comprehensive introduction. The exposition below assumes a quite high degree of regularity for the parameterized density families, which is nevertheless a common situation in practice, especially in the field of machine learning we are interested in.

### 2.1. Definitions and Properties

**Definition** **1.** *A statistical model is a pair $\left(M,p\right)$ where M is an oriented n dimensional smooth manifold and ${\left(p_{\theta }\right)}_{\theta \in M}$ is a parameterized family of probability densities on a measured space (Ω,T,μ) such that, putting $p\left(\theta ,\omega \right)=p_{\theta }\left(\omega \right)$:*

- *For μ-almost all ω∈Ω, the mapping θ↦p(θ,ω) is smooth;*
- *For any θ∈M, there exists an open neighborhood $U_{\theta }$ of θ and an integrable mapping $h:\Omega \to R^{+}$ such that, for any $\xi \in U_{\theta }$, $|\partial_{\theta }p\left(\xi ,\omega \right)|\leq h$;*
- *The mapping $\theta \to p_{\theta }\in L^{1}\left(\Omega ,\mu \right)$ is one-to-one;*
- *The support of $p_{\theta }$ does not depend on θ.*

Assuming *p* never vanishes, one can define the score l:M×Ω→R as:

(6) l(θ,ω)=logp(θ,ω)

For any θ∈M:

(7) $$\int_{\Omega }p_{\theta }\left(\omega \right)d\mu \left(\omega \right)=1$$

Thus, using the fact that the assumptions made on family $p_{\theta }$ allow swapping derivatives and integrals, it becomes:

(8) $$\int_{\Omega }\partial_{i}p\left(\theta ,\omega \right)d\mu \left(\omega \right)=0,\;i=1\ldots n$$

where $\partial_{i}$ denotes the derivative with respect to the *i*-th component of θ in local coordinates. So, the score $l_{\theta }=\log p_{\theta }$ satisfies by (8):

(9) $$E{\left[\partial_{i}l_{\theta }\right]}_{p_{\theta }}=0,\;i=1\ldots n.$$

A simple computation shows that:

(10) $$E\left[\partial_{i}l_{\theta }\partial_{j}l_{\theta }\right]=\int_{\Omega }\frac{\partial_{i}p_{\theta }}{\sqrt{p_{\theta }}}\frac{\partial_{j}p_{\theta }}{\sqrt{p_{\theta }}}d\mu \left(\omega \right)=4\int_{\Omega }\partial_{i}\left(\sqrt{p_{\theta }}\right)\partial_{j}\left(\sqrt{p_{\theta }}\right)d\mu \left(\omega \right),\;i,j=1\ldots n$$

proving that:

(11) $$g_{ij}=E\left[\partial_{i}l_{\theta }\partial_{j}l_{\theta }\right]={\langle \partial_{i}\left(\sqrt{p_{\theta }}\right),\partial_{j}\left(\sqrt{p_{\theta }}\right)\rangle }_{L^{2}\left(\Omega ,\mu \right)}$$

Let *g* be the section of $TM^{*}⊗TM^{*}$ defined by:

(12) $$g=g_{ij}d\theta^{i}⊗d\theta^{j}$$

Now, given any tangent vector $X=X^{i}\partial_{i}\in T_{\theta }M$:

(13) $$\begin{array}{cc} g\left(\theta ;X,X\right)=g_{ij}X^{i}X^{j} & ={\langle \partial_{i}\left(\sqrt{p_{\theta }}\right),\partial_{j}\left(\sqrt{p_{\theta }}\right)\rangle }_{L^{2}\left(\Omega ,\mu \right)}\\ \; & ={\langle X^{i}\partial_{i}\left(\sqrt{p_{\theta }}\right),X^{j}\partial_{j}\left(\sqrt{p_{\theta }}\right)\rangle }_{L^{2}\left(\Omega ,\mu \right)}\\ \; & ={\langle Z,Z\rangle }_{L^{2}\left(\Omega ,\mu \right)} \end{array}$$

with $Z=X^{i}\partial_{i}\left(\sqrt{p_{\theta }}\right).$ Given the assumptions made on the family $p_{\theta }$, *g* is a thus a positive definite symmetric section of TM⊗TM, hence a Riemannian metric on M called the Fisher Information Metric (FIM).

**Remark** **1.** *The mapping $I:\theta \mapsto \sqrt{p_{\theta }}$ embeds M as a submanifold of the unit sphere in $L_{\Omega ,\mu }^{2}$ and the Fisher information metric is just the pullback of the ambient metric in $L_{\Omega ,\mu }^{2}$ with respect to I. However, in machine learning applications, it is common to consider parameter spaces for which the one-to-one assumption for I is non-valid so that g is only positive semidefinite. The study of the rank of the metric in this case is an important research topic.*

It is quite fruitful to consider differential forms on M parameterized by Ω. The starting point is the definition of parameterized degree 0 forms.

**Definition** **2.** *A parameterized 0-form is a mapping f:M×Ω→R satisfying:*

- *For almost all ω∈Ω, the mapping θ∈M→f(θ,ω) is smooth;*
- *For all $\theta_{0}\in \Omega$, and all integers n, there exists a neighborhood $U_{n,\theta_{0}}$ and an integrable positive mapping $h_{n,\theta_{0}}$ such that for all $\theta \in U_{n,\theta_{0}}$ and almost all ω∈Ω: $\left|\partial_{\theta }^{n}f\left(\theta ,\omega \right)\right|\leq h_{n,\theta_{0}}\left(\omega \right).$*

**Proposition** **1.** *Let X be a vector field on TM and f a parameterized 0-form in the previous sense. Then:*

(14) $$X\left(E\left[f\right]\right)=E\left[X\left(f\right)\right]+E\left[fX(l)\right]$$

*with l(θ,ω)=logp(θ,ω)n.*

**Proof.**  $E\left[f\right]$ is a degree 0 form on TM. If ψ is the flow of *X*, then:

(15) $$\psi^{★}E\left[f\right]=\int_{\Omega }f\left(\psi (t,\theta ),\omega \right)p\left(\psi (t,\theta ),\omega \right)d\mu \left(\omega \right)$$

The assumptions made on *f* allowing the swapping of derivatives and integrals, so:

(16) $${\frac{\partial }{\partial t}}_{t=0}E\left[f\right]=\int_{\Omega }\partial_{\theta }f\left(\theta ,\omega \right)X\left(\theta \right)p\left(\theta ,\omega \right)d\mu \left(\omega \right)+\int_{\Omega }f\left(\theta ,\omega \right)\frac{\partial_{\theta }p\left(\theta ,\omega \right)}{p(\theta ,\omega )}p\left(\theta ,\omega \right)d\mu \left(\omega \right)$$

□

**Remark** **2.** *Applying Proposition 1 to the constant function f=1 yields $E\left[X(l)\right]=0$, a result already known by Equation (9)*

A parameterized degree *k* differential form on TM can be defined readily by requiring that the coefficients of the elementary forms $d\theta_{i_{1}}\wedge \cdots \wedge d\theta_{i_{k}}$ be parameterized differential forms of degree 0.

**Proposition** **2.** *Let α be a degree k parameterized differential form on TM. Then:*

(17) $$dE\left[\alpha \right]=E\left[d\alpha \right]+E\left[dl\wedge \alpha \right]$$

**Proof.**  It is enough to consider a form $\alpha \left(\theta ,\omega \right)=f\left(\theta ,\omega \right)d\theta_{i_{1}}\wedge \cdots \wedge d\theta_{i_{k}}.$ Then:

(18) $$dE\left[\alpha \right]\left(\theta \right)=\sum_{j=1}^{n}E\left[\partial_{\theta_{j}}f\right]d\theta_{j}\wedge d\theta_{i_{1}}\wedge \cdots \wedge d\theta_{i_{k}}+\sum_{j=1}^{n}E\left[f\partial_{\theta_{j}}l\right]d\theta_{j}\wedge d\theta_{i_{1}}\wedge \cdots \wedge d\theta_{i_{k}}$$

since:

(19) $$\begin{array}{cc} \; & d\alpha =\sum_{j=1}^{n}\left(\partial_{\theta_{j}}f\right)d\theta_{j}\wedge d\theta_{i_{1}}\wedge \cdots \wedge d\theta_{i_{k}} \end{array}$$

(20) $$\begin{array}{cc} \; & dl\wedge \alpha =\sum_{j=1}^{n}f\left(\partial_{\theta_{j}}l\right)d\theta_{j}\wedge d\theta_{i_{1}}\wedge \cdots \wedge d\theta_{i_{k}} \end{array}$$

the claim follows. □

Proceeding the same way as in Proposition 1, and using Cartan’s homotopy formula, we obtain:

**Proposition** **3.** *Let X be a vector field on TM and α a degree k parameterized differential form. Then*

(21) $$L_{X}\left(E\left[\alpha \right]\right)=E\left[i_{X}d\alpha \right]+E\left[di_{X}\alpha \right]+E\left[\left(i_{X}dl\right)\wedge \alpha \right]$$

When α=dl, Equation (21) reads as:

(22) $$L_{X}E\left[dl\right]=E\left[i_{X}d^{2}l\right]+E\left[d\left(i_{X}dl\right)\right]+E\left[\left(i_{X}dl\right)\wedge dl\right]$$

Since $E\left[dl\right]=0$, it becomes:

(23) $$E\left[d\left(i_{X}dl\right)\right]=-E\left[\left(i_{X}dl\right)\wedge dl\right]$$

Given two vector fields X,Y:

(24) $$i_{Y}E\left[\left(i_{X}dl\right)\wedge dl\right]=E\left[\left(i_{X}dl\right)\left(i_{Y}dl\right)\right]=g\left(X,Y\right)$$

with *g* the Fisher metric. Thus:

**Proposition** **4.**
(25) $$g\left(X,Y\right)=-E\left[i_{Y}d\left(i_{X}dl\right)\right]$$

**Remark** **3.** *In coordinates, $i_{Y}d\left(l_{X}dl\right)=\partial_{ij}X^{j}Y^{i}+\partial_{j}l\partial_{i}X^{j}Y^{i}$, and after taking the expectation:*

(26) $$g\left(X,Y\right)=-E\left[\partial_{ij}l\right]X^{j}Y^{i}$$

*This is a well-known result in the $R^{n}$ case.*

Let ∇ be an affine connection on TM. The same computation as above yields:

**Proposition** **5.** *Let X be a vector field on TM and α a degree k parameterized differential form. Then:*

(27) $$\nabla_{X}E\left[\alpha \right]=E\left[\nabla_{X}\alpha \right]+E\left[\left(i_{X}dl\right)\wedge \alpha \right]$$

When α=dl, we recover $E\left[\nabla_{X}dl\right]\left(Y\right)=-g\left(X,Y\right)$, showing that while the parameterized Hessian ∇dl depends on the connection ∇, it is not the case of its expectation. When $\Omega =M=R^{n},\mu =dx_{1}dx_{2}\ldots dx_{n}$, the Fisher metric is known to be twice the second order term in the Taylor expansion of the Kullback–Leibler divergence, which can be proved easily by iterating derivatives. More generally, let ∇ be a connection and let θ:]−ϵ,ϵ[→M,ϵ>0 be a smooth curve with $\theta_{0}=\theta \left(0\right),X=\theta^{\prime }\left(0\right)$. Recall that the Kullback–Leibler divergence between two probability densities p,q is defined as:

(28) $$\mathrm{KL}\left(p,q\right)=E_{p}\left[\log\left(p/q\right)\right]=\int \log\left(\frac{p(x)}{q(x)}\right)p\left(x\right)dx$$

The mapping:

(29) $$t\in ]-\epsilon ,\epsilon [\mapsto \xi \left(t\right)=\mathrm{KL}\left(p_{\theta_{0}},p_{\theta (t)}\right)=E_{p_{\theta_{0}}}\left[l_{\theta_{0}}\left(t\right)-l_{\theta }\left(t\right)\right]$$

is smooth, so Taylor formula applies for *t* close enough to 0:

(30) $$\xi \left(t\right)=\sum_{i=1}^{n}\frac{\xi^{\left(i\right)}\left(0\right)}{i!}t^{i}+o\left(t^{n}\right)$$

With:

(31) $$\xi^{\left(i\right)}\left(0\right)=E_{p_{\theta_{0}}}\left[\underset{i\;\mathrm{times}\;}{\underbrace{X\left(X\left(\ldots X(l)\right)\right)}}\right]=E_{p_{\theta_{0}}}\left[\underset{i-1\;\mathrm{times}\;}{\underbrace{X\left(X\left(\ldots dl(X)\right)\right)}}\right]$$

If the curve t→θ(t) is a geodesic for ∇, then:

(32) $$X\left(dl(X)\right)=\left(\nabla_{X}dl\right)\left(X\right)+dl\left(\nabla_{X}X\right)=\left(\nabla_{X}dl\right)\left(X\right)$$

And, by recurrence:

(33) $$\xi^{\left(i\right)}\left(0\right)=E_{p_{\theta_{0}}}\left[\left(\nabla_{X}^{\left(i-1\right)}dl\right)\right]\left(X\right).$$

The first derivative $\xi^{\left(1\right)}\left(0\right)$ is readily computed as:

(34) $$-E\left[dl_{\theta_{0}}\right]\left(X\right)=0.$$

The second derivative $\xi^{\left(2\right)}\left(0\right)$ can be obtained using ∇ as:

(35) $$-E\left[\nabla_{X}dl_{\theta_{0}}\right]\left(X\right)=g_{\theta_{0}}\left(X,X\right).$$

Since *g* is symmetric, $g\left(X,Y\right)=\left(g(X+Y,X+Y)-g(X-Y,X-Y)\right)/4$, thus (35) characterizes *g* as $\theta_{0}.$ Higher-order terms can be computed by repeatedly applying Proposition 5 and are expressed thanks to the quantities:

(36) $$E\left[\left(i_{X}dl\right)\wedge \nabla_{X}^{\left(i\right)}dl\right]\left(X\right).$$

An interesting case occurs when the Fisher metric is non-degenerate and $\nabla^{\mathrm{lc}}$ is its associated Levi-Civita connection. Normal coordinates at $\theta_{0}$, denoted by $x^{i},\;i=1\ldots N$, are given by taking an orthonormal basis, with respect to the Fisher metric, $\left(v_{1},\ldots ,v_{N}\right)$ and letting [28] (p. 72):

(37) $$x^{i}\left(\exp_{\theta_{0}}t^{j}v_{j}\right)=t^{i}$$

Using the $x^{i},i=1\ldots N$ system of coordinates in place of θ, and noting that $\theta_{0}$ corresponds to the origin in normal coordinates, the KL divergence can be approximated at order 2 by:

(38) $$\mathrm{KL}\left(p_{0},p_{x}\right)=\frac{1}{2}x^{i}x^{j}$$

where $x=\left(x^{1},\ldots ,x^{N}\right).$

### 2.2. The Fisher Information in Machine Learning

In machine learning applications, when the output is a probability distribution, then the Kullback–Leibler divergence is a natural measure for goodness-of-fit. Assuming that the database is given in the form of an iid sample of couples ${\left(X_{i},Y_{i}\right)}_{i=1\ldots N}$, then one can introduce the error function:

(39) $$E\left(W\right)=\sum_{i=1}^{N}\mathrm{KL}\left(Y_{i},N\left(X_{i},W\right)\right)$$

That may be approximated by:

(40) $$\tilde{E}\left(W\right)=-\sum_{i=1}^{N}\frac{1}{2}g\left(Y_{i};\vec{Y_{i}N\left(X_{i},W\right)},\vec{Y_{i}N\left(X_{i},W\right)}\right)$$

where the notation $\vec{PQ}$ stands for the tangent vector at *P* such that a geodesic (for $\nabla^{\mathrm{lc}}$) θ with $\theta \left(0\right)=P,\theta^{\prime }\left(0\right)=\vec{PQ}$ is such that θ(1)=Q. Taking the derivative with respect to *W* yields:

(41) $$\frac{\partial \tilde{E}}{\partial W}=-\sum_{i=1}^{N}g\left(Y_{i};\frac{\partial N\left(X_{i},W\right)}{\partial W},\vec{Y_{i}N\left(X_{i},W\right)}\right)$$

with $\frac{\partial N\left(X_{i},W\right)}{\partial W}$ being a tangent vector at $Y_{i}$.

We recall the musical isomorphism $♭:TM\to TM^{★}$ defined by:

(42) $$X^{♭}\left(Y\right)=g\left(X,Y\right)$$

and use it to rewrite (41) as:

(43) $$\frac{\partial \tilde{E}}{\partial W}=-\sum_{i=1}^{N}{\left(\frac{\partial N\left(X_{i},W\right)}{\partial W}\right)}^{♭}\left(\vec{Y_{i}N\left(X_{i},W\right)}\right)$$

In this form, having a critical point of the energy $\tilde{E}$ with respect to *W* is equivalent to the vanishing of a totally symmetric multilinear form on $TM⊕TM^{★}$, the generalized tangent bundle of M.

Finally, if ψ:N→M is a smooth mapping, one can take the pullback the Fisher metric on M to obtain a semi-definite symmetric bilinear form on N:

(44) $$\psi^{★}g\left(\eta ;X,Y\right)=g\left(\psi \left(\eta \right);\psi^{\prime }\left(\eta \right)X,\psi^{\prime }\left(\eta \right)Y\right)$$

When ψ is an embedding, $\psi^{★}g$ is a Fisher metric on N with $p_{\psi (\eta )},\eta \in N$ as underlying densities. This is the case considered in [25].

As an example of a pullback metric, we are going to investigate the case of the von Mises–Fisher distribution (VMF) on $S^{n-1}$ with density:

(45) $$p_{\kappa ,\mu }\left(x\right)=\frac{\kappa^{n/2-1}}{{\left(2\pi \right)}^{n/2}I_{n/2-1}\left(\kappa \right)}\exp\left(\kappa \langle x,\mu \rangle \right)$$

where κ≥0 is the concentration parameter, $\mu \in S^{n-1}$ is the location parameter and $I_{k}$ is the modified Bessel function of the first kind of order *k*. The Fisher metric in the embedding space $R^{n}$ can be deduced from the second moment $E\left[xx^{t}\right]$ since $l_{\kappa ,\mu }=\log\left(p_{\kappa ,\mu }\right)=f\left(\kappa \right)+\langle x,\mu \rangle$. If κ is assumed to be constant, then:

(46) $$E\left[\partial_{\mu }l_{\kappa ,\mu }l_{\kappa ,\mu }^{t}\right]=E\left[xx^{t}\right]$$

Although the expression for $E\left[xx^{t}\right]$ has been given in [29], we present here an alternative proof based on the fact that for any integer *n*, $S^{n-1}$ is a suspension of $S^{n-2}$. If $x=\left(x_{1},\ldots ,x_{n}\right)$, then $xx^{t}$ is a matrix whose (i,j) entry is $x_{i}x_{j}$. By the rotation invariance of the VMF, μ can be selected as the first vector of an orthonormal basis, with respect to which *x* is expressed in components as $x=\left(x_{1},\ldots ,x_{n}\right)$. If we specialize the first component, then, if i≠1,j≠1:

(47) $$\int_{S^{n-1}}x_{i}x_{j}p_{\kappa ,\mu }\left(x\right)dx=c_{\kappa }\int_{0}^{\pi }\exp\left(\cos\theta \right)\sin^{n-2}\left(\theta \right)\int_{S^{n-2}}\xi_{i}\xi_{j}d\sigma_{n-2}\left(\xi \right)$$

with $x_{i}=\sin\theta \xi_{i},i=1\ldots n-1$ and $\sigma_{n-2}$ the Lebesgue measure on $S^{n-2}$. If i≠j, then the integral vanishes by symmetry, otherwise:

(48) $$\begin{array}{cc} \int_{S^{n-2}}\xi_{i}\xi_{j}d\sigma_{n-2}\left(\xi \right) & =\int_{0}^{\pi }\cos^{2}\left(\psi \right)\sin^{n-3}\left(\psi \right)\int_{S^{n-3}}d\sigma_{n-3}d\psi \\ \; & =\int_{0}^{\pi }\cos^{2}\left(\psi \right)\sin^{n-3}\left(\psi \right)d\psi A\left(S^{n-3}\right) \end{array}$$

with $A\left(S^{n-3}\right)$ the area of the n−3-sphere, which is given by the general relation:

(49) $$A\left(S^{n}\right)=\frac{2\pi^{\frac{n+1}{2}}}{\Gamma \left(\frac{n+1}{2}\right)}$$

Now, observing that [30]:

(50) $$\int_{0}^{\pi }\cos^{2}\left(\psi \right)\sin^{n-3}\left(\psi \right)d\psi =B\left(\frac{3}{2},\frac{n}{2}-1\right)$$

with *B* the beta function, the overall expression becomes, after using (49):

(51) $$\begin{array}{cc} \; & \frac{{\left(2\pi \right)}^{n/2}\Gamma \left(\frac{n}{2}-1\right)I_{n/2}\left(\kappa \right)\kappa^{n/2-1}}{\kappa^{n/2}\Gamma \left(n/2-1\right){\left(2\pi \right)}^{n/2}I_{n/2-1}\left(\kappa \right)}\\ \; & =\frac{1}{\kappa }\frac{I_{n/2}\left(\kappa \right)}{I_{n/2-1}\left(\kappa \right)} \end{array}$$

When i=j=1, then the expression for the second moment becomes:

(52) $$\begin{array}{cc} \; & \int_{0}^{\pi }\exp\left(\kappa \cos\theta \right)\cos^{2}\left(\theta \right)\sin^{n-2}\left(\theta \right)d\theta A\left(S^{n-2}\right)=\\ \; & \int_{0}^{\pi }\exp\left(\kappa \cos\theta \right)\left(1-\sin^{2}\left(\theta \right)\right)\left(\theta \right)\sin^{n-2}\left(\theta \right)d\theta A\left(S^{n-2}\right) \end{array}$$

The integral is a difference of two terms, each of which can be simplified as before to yield:

(53) $$\left(1-\frac{n}{\kappa }\right)\frac{I_{n/2}\left(\kappa \right)}{I_{n/2-1}\left(\kappa \right)}$$

This procedure can easily be applied to an arbitrary moment, each of the integral involved being expressible using $I_{n}$ and the Beta function.

**Remark** **4.** *Since μ is not a parameterization of the unit sphere, the Fisher metric defined that way is related to an ambient metric in $R^{n}$, defined only on the unit sphere.*

An obvious embedded dimension n−2 submanifold of $S^{n-1}$ is obtained by taking a unit vector ν and computing the intersection of $S^{n-1}$ with an hyperplane H defined by:

(54) x∈H⇔〈x,ν〉=αα∈[0,1]

An elementary computation proves that the intersection locus is a n−2 sphere contained in H:

(55) $${\left|x-\alpha \nu \right|}^{2}=1-\alpha^{2}$$

Without loss of generality, ν can be taken as $\left(\begin{array}{cccc} 1 & 0 & \ldots & 0 \end{array}\right)$ and the embedding can be written easily as:

(56) $$\left(\begin{array}{ccc} x_{1} & \ldots & x_{n-1} \end{array}\right)\mapsto \left(\begin{array}{cccc} \alpha & \lambda x_{1} & \ldots & \lambda x_{n-1} \end{array}\right),\;\lambda =\sqrt{1-\alpha^{2}}$$

The pullback metric is just the original one scaled by $1-\alpha^{2}$. The loss functions related to the VMF distribution are discussed in [31].

## 3. Pullback Bundles

In this section, a neural network with weights *W* is a mapping $N\left(\cdot ,W\right):I\to O$, where I (i.e., O) is the input (i.e., output) manifold of dimension *n* (i.e., *m*). Both manifolds are assumed to be smooth, and also the mapping $N_{W}.$ This last assumption is valid when the activation functions are smooth, which is the case for sigmoid functions, but not for the commonly used ReLu function. However, smooth approximations to the ReLu are easy to construct with an arbitrary degree of accuracy, so the framework introduced below can be still applied.

As mentioned in the introduction, O is further assumed to be a statistical model 1 with Fisher metric g. This setting is the one of a neural network whose output is a random variable with conditional density in a family $p_{\theta },\theta \in O$.

When the weights are kept fixed, the only free parameters are the inputs and the network is fully described by the mapping:

(57) $$\left\{\begin{array}{cc} N\left(\cdot ,W\right): & I\to O\\ \; & x\mapsto N\left(\cdot ,W\right)=p_{\theta (x)} \end{array}\right.$$

For the ease of notation, the mapping $N\left(\cdot ,W\right)$ will be abbreviated by $N_{W}\left(\cdot \right)$. When the activation functions in the network are smooth, $N_{W}\left(\cdot \right)$ is a smooth mapping and its derivative will be denoted by $dN_{W}\left(\ldots \right)$. With this convention, the pullback metric of *g* by $N_{W}\left(\cdot \right)$, denoted $\tilde{g}$, is defined by:

(58) $$\tilde{g}\left(X,Y\right)=g\left(dN_{W}\left(X\right),dN_{W}\left(Y\right)\right)$$

**Figure 1:**
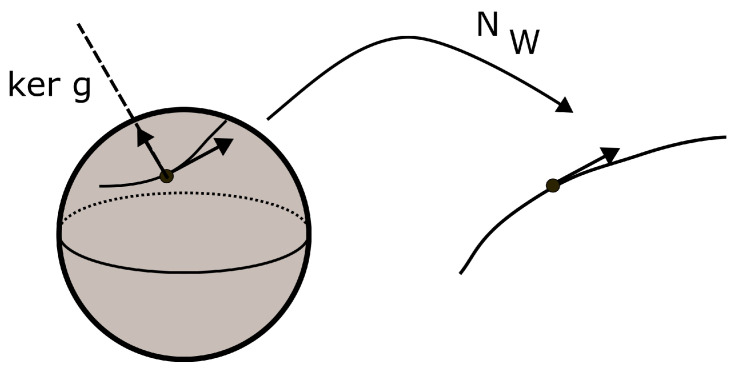
Kernel of the pullback metric.

Unless the network N is a decoder, $\tilde{g}$ is generally degenerated and does not provide I with a Riemannian structure, so an ambient metric *h* on I is assumed to exist. The triple $\left(I,h,\tilde{g}\right)$ is called the data manifold of the network. The kernel of $\tilde{g}$, denoted $\ker\tilde{g}$, is the distribution in TI consisting of vectors *X* such that $\tilde{g}\left(X,\cdot \right)$ is the zero mapping. At a point x∈I, the vectors in $T_{x}I$ belonging to $\ker\tilde{g}$ give directions in which the output of the network will not change up to order 1. Figure 1 represents the case of a one dimensional output space and a 2-sphere input space. Since the dimension of the output is less than the one of the input, some moves in the data manifold will not induce any change at the output.

Unless the dimension of $\ker\tilde{g}$ is constant, this distribution does not define a foliation. However, this is true locally in the neighborhood of points in I such that $dN_{W}\left(\cdot \right)$ has maximal rank. Finally, if $E\overset{\pi }{\to }O$ is an *r*-vector bundle on O, then its pullback by $N_{W}\left(\cdot \right)$ will be denoted in short by $E^{N_{W}}.$ We recall that if *E* has local charts:

$$\left(V_{i},\xi_{i}\right),\;\xi_{i}:V_{i}\times R^{r}\to \pi^{-1}\left(V_{i}\right),\;i\in I$$

and I has local charts $\left(U_{j},\phi_{i}\right),\;j\in J$, then $E^{N_{W}}$ has local charts:

(59) $$\begin{array}{cc} \; & \left(W_{ji}=U_{j}\cap N_{W}^{-1}\left(V_{i}\right),\psi_{ji}\right),\;\psi_{ji}:W_{ji}\to W_{ji}\times R^{r}\\ \; & \psi_{ji}\left(x\right)=\xi_{i}\circ f\circ \phi_{j} \end{array}$$

The pullback bundle enjoys a universal property that is in fact the main reason for introducing it in our context.

**Proposition** **6.** *Let $\left(\tilde{E},\tilde{\pi },I\right)$ (i.e., $\left(E,\pi ,O\right)$) be a vector bundle on I (i.e., O)). For any bundle morphism $\left(\eta_{1},\eta_{0}\right)$, there exists a unique bundle morphism $\left({\tilde{\eta }}_{1},Id\right)$ such that the following diagram commutes:*

(60)

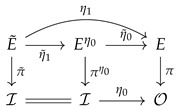


*where $\pi^{\eta_{0}}:\left(x,v\right)\mapsto x$ and ${\tilde{\eta }}_{0}:\left(x,v\right)\mapsto \left(\eta_{0}\left(x\right),v\right).$*

This proposition is a classical one and its proof can be found in many textbooks. The one we give below is very simple, using only local charts.

The above construction is constructive and thus gives a practical mean of computation. For a network with fixed weights, e.g., a trained one, the derivative $dN_{W}$ can be efficiently computed by back propagation, so the bundle morphism: (61)
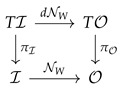

has a practical meaning.

Introducing the pullback bundle gives the diagram: (62)
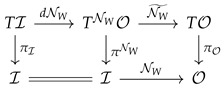


The bundle mapping $dN_{W}$ to $T^{N_{W}}O$ is then the association:

(63) $$\left(x,v\right)\in R^{m}\times R^{n}\mapsto \left(x,dN_{W}\cdot v\right)\in R^{n}\times R^{n}$$

The pullback bundle is thus a mean of representing the action of the network on tangent vectors to the data manifold. As an example, the construction of adversarial attacks given in [32,33] can be revisited in this context, extending it to the general setting of network with manifold inputs.

The general problem of building an adversarial attack is, informally, to find, for an input point in the data manifold, a direction in which a perturbation will have the most important effect on the output, hopefully fooling the network. Following [33], we define:

**Definition** **3.** *Let h be a Riemannian metric on the input space. An optimal adversarial attack at x∈I with budget ϵ>0 is a solution to:*

(64) $$\max_{v\in T_{x}I,h\left(v,v\right)\leq \epsilon }\tilde{g}\left(v,v\right)$$

Using (38), this optimization program can be viewed as a local approximation to the one based on the Kullback–Leibler divergence:

**Definition** **4.** *A Kullback–Leibler optimal adversarial attack at x∈I with budget ϵ>0 is a solution to:*

(65) $$\max_{y\in I,h(x,y)\leq \epsilon }\mathrm{KL}\left(N\left(x,W\right),N\left(y,W\right)\right)$$

The metric *g* on TO can be pulled back to $T^{N_{W}}$ by letting:

(66) $$g^{N_{W}}\left(x;v,v\right)=g\left(f(x);v,v\right)$$

Due to the special form of the criterion, the optimal point is on the boundary, so that finally, the optimal adversarial attack problem may be formulated as:

**Definition** **5.** *An optimal adversarial attack at x∈I with budget ϵ>0 is a solution to:*

(67) $$\max_{v\in UT_{x}I}\epsilon^{2}g^{N_{W}}\left(dN_{W}v,dN_{W}v\right)$$

Where UTI stands for the unit sphere bundle with respect to the metric h. Please note that due to bilinearity, the problem can be solved for ϵ=1, then let the optimal vector be scaled by the original ϵ. From standard linear algebra, if $G_{x}$ is the matrix of the bilinear form $g^{N_{W}}$ at *x* and $H_{x}$ the one of *h*, then one can find unitary matrices A,B and diagonal matrices Λ,Σ such that:

(68) $$H_{x}=A^{t}\Lambda A,\;G_{x}=B^{t}\Sigma B$$

Any vector *v* in $UT_{x}I$ can be written as:

(69) $$V=A^{t}\Lambda^{-1/2}w,w^{t}w=1$$

So that, finally, the original problem can be rewritten as:

(70) $$\max_{w,w^{t}w=1}w^{t}M^{t}Mw,\;M=\Sigma^{1/2}Bd_{x}N_{W}A^{t}\Lambda^{-1/2}$$

which is solved readily by taking *w* to be the unit eigenvector of *M* associated with the largest eigenvalue. This is the solution found in [33] when $H_{x}=\mathrm{Id}.$

In many cases, as the above example indicates, it is more convenient to work uniquely in the input space, thus justifying the introduction of the pullback bundle $T^{N_{W}}O.$ From now, we are going to adopt this point of view.

**Remark** **5.** *Please note that a section in $T^{N_{w}}I$ is generally not related to a section of the form (63) in either TO or TI due to the fact that $d_{x}N_{W}$ may not be a monomorphism or an epimorphism. The next proposition gives condition for the existence of global sections in TO associated with global sections in $T^{N_{W}}O.$*

**Proposition** **7.** *In the case of a decoding network, when $N_{W}$ is an embedding, there is a natural embedding of bundles $\begin{array}{ccc} TI & \overset{i}{↪} & T^{N_{W}}O \end{array}$ such that the image of (x,v) is $\left(x,dN_{W}v\right)$. The pullback bundle then splits as:*

(71) $$T^{N_{W}}O=i\left(TI\right)⊕F$$

*where F has rank n−m.*

Be careful that in this case, a section of the pullback bundle will not define a global section in TO since some points of the output space may have no preimage by $N_{W}.$ However, by the extension lemma [34] (Lemma 5.34, p. 115), local (global if $N_{W}I$ is closed) smooth vector fields on TO exist, extending it.

**Proof.**  If $N_{W}$ is an embedding, $N_{W}\left(I\right)$ is a submanifold of O and in an adapted chart, a vector field in $TN_{W}\left(I\right)$ can be written as $v=\sum_{i=1}^{n}v^{i}\partial_{i}$, where the $\partial_{i},i=1\ldots n$ are the first *n* coordinate vector fields. It thus pulls back to a section $\tilde{v}$ of the same form in $T^{N_{W}}O$. Now, since $dN_{W}$ is injective, $\tilde{v}$ is the image of a unique section in TI, hence the claim. □

**Proposition** **8.** *If $\ker dN_{W}$ has constant rank r, then there exists a splitting $TI=\ker dN_{W}⊕F$, $T^{N_{W}}O=im\;dN_{W}⊕G$ and bundle isomorphism $F\to im\;dN_{W}$ that coincides with $dN_{W}$ on the fibers.*

**Proof.**  By Theorem 10.34, [34] (p. 266), $\ker dN_{W}$ is a subbundle of TI and $\mathrm{im}dN_{W}$ a subbundle of $T^{N_{W}}O$. In local charts, the morphism $dN_{W}$ gives rise to the decomposition:

(72) $$\begin{array}{ccc} \ker dN_{W}⊕R^{r} & \overset{dN_{W}}{\to } & \mathrm{im}dN_{W}⊕R^{m-r} \end{array}$$

with $dN_{W}$ an isomorphism where restricted to $R^{r}.$ Passing to local sections yields the result. □

An important case is the one of submersions, corresponding to encoders in machine learning. In this case, r=m and $dN_{W}$ establishes a bundle isomorphism between *F* and $T^{N_{W}}O.$ The pullback of Fisher–Rao metric *g* on TO gives rise to a metric $g^{N_{W}}$ on $T^{N_{W}}O$, but only to a degenerate metric on TI that can, nevertheless, be quite well understood, as indicated below.

**Definition** **6.** *On the input bundle TI, the symmetric tensor $\tilde{g}$ is defined using the splitting $TI=\ker dN_{W}⊕F$, by:*

(73) $$\begin{array}{cc} \; & g\left(X,Y\right)=0,\;X\in \ker dN_{W},\;Y\in TI\\ \; & g\left(X,Y\right)=g^{N_{W}}\left(dN_{W}X,dN_{W}Y\right),\;X,Y\in F \end{array}$$

**Proposition** **9.** *There exists a symmetric (1,1)-tensor on I, denoted by *Θ*, such that, for any tangent vectors (X,Y)∈TI:*

(74) $$h\left(\Theta X,Y\right)=\tilde{g}\left(X,Y\right)$$

**Proof.**  From standard linear algebra, there exists an adjoint ${}^{t}dN_{W}$ to $dN_{W}$, defined by:

(75) $$g^{N_{W}}\left(dN_{W}v,dN_{W}v\right)=h\left({}^{t}dN_{W}v,dN_{W}v\right)$$

with, in local coordinates:

(76) $${}^{t}N_{i}^{j}=h^{il}N_{l}^{k}g_{ij}^{N_{W}}$$

where *N* (i.e., ${}^{t}N$ ) is the matrix associated with $dN_{W}$ (i.e., ${}^{t}dN_{W}$) and, as usual, $h^{il}={\left(h^{-1}\right)}_{il}.$ The (1,1)-tensor Θ is then the product ${}^{t}dN_{W}dN_{W}.$ □

**Remark** **6.** *Θ is defined even if $dN_{W}$ is not full rank.*

**Remark** **7.** *All the relevant information concerning $dN_{W}$ is encoded in Θ. As a consequence, the geometry of an encoder is described by this tensor, hence also the one of an encoder–decoder block.*

**Remark** **8.** *The tensor *Θ* has expression $g_{pj}N_{i}^{j}N_{k}^{p}$ in a local orthonormal frame, hence is symmetric.*

**Definition** **7.** *Let *∇* be a connection on TI. Its dual connection $\nabla^{★}$ is defined by the next equation:*

(77) $$\nabla_{Z}h\left(X,Y\right)=h\left(\nabla_{Z}X,Y\right)+h\left(X,\nabla_{Z}^{★}Y\right)$$

*where Z is any tangent vector in TI and X,Y are vector fields.*

**Definition** **8.** *A (1,1)-tensor *Θ* is said to satisfy the gauge equation [35] if, for all tangent vectors Z:*

(78) $$\nabla_{Z}^{★}\Theta =\Theta \nabla_{Z}$$

**Proposition** **10.** *If *Θ* satisfies the gauge Equation (78), then the (0,2)-tensor defined by:*

(79) $$\left(X,Y\right)\mapsto h\left(\Theta X,Y\right)$$

*is *∇* parallel.*

**Proof.**  For any vector fields X,Y, and any tangent vector *Z*:

(80) $$\begin{array}{cc} \; & \nabla_{Z}^{★}h\left(\Theta X,Y\right)=h\left(\nabla_{Z}^{★}\Theta X,Y\right)+h\left(\Theta X,\nabla_{Z}Y\right)\\ \; & =h\left(\Theta \nabla_{Z}X,Y\right)+h\left(\Theta X,\nabla_{Z}Y\right) \end{array}$$

hence the claim. □

Θ, being symmetric, admits a diagonal expression in a local orthonormal local frame $\left(X_{1},\ldots ,X_{n}\right)$. When there exists a connection ∇ such that $\nabla_{Z}^{★}\Theta X=\Theta \nabla_{Z}X$ for any vector fields X,Z, parallel transport of the $X_{i},i=1\ldots n$ shows that the eigenvalues are constant and the eigenspaces preserved. The existence of a solution to the gauge equation thus greatly simplifies the study of an encoder, as a local splitting of the input manifold exists. The reader is referred to [35] for more details. In fact, the tensor Θ is defined even if for general networks and the splitting may exist in this setting. This is the case when the rank of $dN_{W}$ is locally constant, hence when it is maximal. A practical computation of Θ can be obtained through the singular value decomposition, as Proposition (74) indicates. A numerical integration of the distribution given by the first singular vectors gives rise to a local system of coordinates, defining in turn a connection satisfying the gauge equation (the existence of a global solution has a cohomological obstruction that is outside the scope of this paper).

Finally, we introduce below a construction that takes into account the weight influence. As mentioned in Section 2, the derivative of the network with respect to its weights is adequately described as a 1-form, thus a section of $T^{★}O.$ In fact, when the inner layers of the network are manifolds, the parameters are no longer real values and a suitable extension has to be introduced. One possible approach is to take a connection ∇ on the layer manifold L. Considering a point p∈L, the exponential $\exp^{\nabla }$ defines a local chart centered at p. Given a point *q* in the injectivity domain of $\exp^{\nabla }$, one can obtain its coordinates as $\log_{p}^{\nabla }q=\vec{pq}$ and the activation of a neuron with input *q* as $\alpha \left(\vec{pq}\right)$, with α a 1-from in $T^{★}L.$ In this general setting, a manifold neuron will be defined by its input in an exponential chart, a 1-form corresponding to the weights in the Euclidean setting and an activation function. Its free parameters are thus a couple $\left(q,\alpha \right)\in TL⊕T^{★}L.$ This particular vector bundle is known as the generalized tangent bundle.

Recalling (43), it is worth to study the pullback of the generalized bundle $TO⊕T^{★}O$. The generalized pullback bundle is then $T^{N_{W}}O⊕T^{★N_{W}}O$, whose local sections are generated by the pullback local sections of the form:

(81) $$\left(x,v\left(N_{W}\left(x\right)\right),\alpha \left(N_{W}\left(x\right)\right)\right)$$

Please note that the pullback can be performed on any layer, internal or input. Most of the previous derivations can be carried out on the generalized bundle, which must be thus considered as a general, yet tractable framework for XAI.

## 4. A Numerical Example

In this example, the input data are the handwritten digits from the MNIST database. A neural network with the next architecture was coded in torch 2 and trained on the dataset:

- First layer: convolutional, kernel size of 3, nonlinearity sigmoid;
- Second layer: convolutional, kernel size of 3, nonlinearity sigmoid;
- Pooling layer;
- Two linear layers;
- Softmax layer.

The input metric is Euclidean, the output one is the Fisher metric of the multinomial distribution with ten classes, that is given by the matrix:

(82) $$\left(\begin{array}{cccc} p_{1}^{-1} & 0 & \ldots & 0\\ 0 & p_{2}^{-1} & \ddots & 0\\ \vdots & \vdots & \vdots & \vdots \\ 0 & \ldots & 0 & p_{9}^{-1} \end{array}\right)+\frac{1}{p_{10}}\left(\begin{array}{ccc} 1 & \ldots & 1\\ \vdots & \vdots & \vdots \end{array}\right)$$

**Figure 2:**
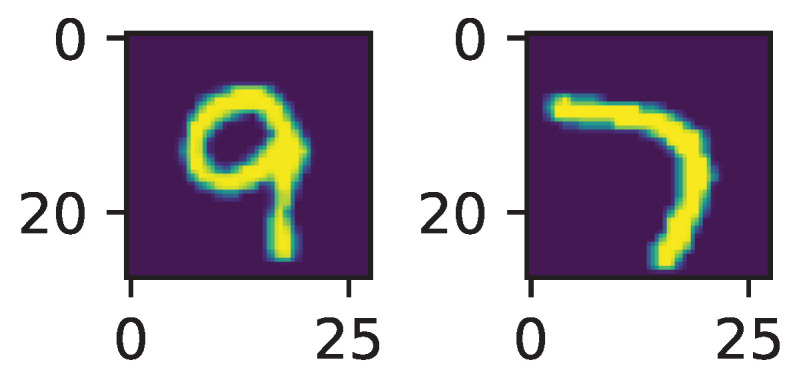
Samples with the highest rotation rate.

Since the output space has dimension 9, the pullback bundle also has dimension 9. At an input point *x*, a point in the pullback bundle is a couple (x,v) with *v* a vector from $R^{9}$ at output point $N_{W}\left(x\right)$. On the other hand, the image of the input tangent bundle (simply a vector space in our case) has points $\left(x,dN_{W}u\right)$ with *u* an input vector. We are thus considering a bundle mapping $\left(x,dN_{W}u\right)\mapsto \left(x,dN_{W}u\right)$ where the right-hand term has values in the pullback bundle, equipped with the output Fisher metric. Tensor Θ is computed via singular value decomposition, already implemented in torch. We selected the rotation rate of the singular vector associated with the largest singular value as an indicator of the complexity of the decision process in the neighborhood of an input point. The code was adapted from https://github.com/eliot-tron/CurvNetAttack (accessed on 12 September 2023). A detection of outliers from a sample of 1000 points was performed. A visual analysis reveals that they correspond to poorly drawn digits, as indicated in Figure 2 where the two digits with the highest curvature indicator are plotted:

The first one is labeled “9”, which is quite obvious for a human operator, although the final stroke is vertical, while the second is labeled “7”, easily confused with a “1”.

## 5. Conclusions and Future Work

In this paper, several important constructions originating from information geometry were surveyed and some new ones introduced. The pullback bundle on a layer allows to describe the behavior of a network with respect to the Fisher information metric, and a simple description can be obtained when a gauge equation is satisfied. One important feature of this construction is its ability to fit in a general framework where layers take their inputs on a manifold.

Future work involves a companion paper describing computational procedures and examples from real case studies. An study of the properties of the pullback generalized bundle is also in progress. Finally, the case of networks with non constant rank $dN_{W}$ must be considered. It is believed that they give rise to singular foliations.

## Data Availability

Not applicable.
